# A comparison of point-tracking algorithms in ultrasound videos from the upper limb

**DOI:** 10.1186/s12938-023-01105-y

**Published:** 2023-05-24

**Authors:** Uriel Magana-Salgado, Praneeth Namburi, Micha Feigin-Almon, Roger Pallares-Lopez, Brian Anthony

**Affiliations:** 1 Department of Mechanical Engineering, MIT, Cambridge, MA 02139 USA; 2 Mechanical Engineering Graduate Program, MIT, Cambridge, MA 02139 USA; 3 Institute for Medical Engineering and Science, Massachusetts Institute of Technology, 77 Massachusetts Ave, 12-3211, Cambridge, MA 02139 USA; 4 MIT.Nano Immersion Lab, MIT, Cambridge, MA 02139 USA

**Keywords:** Ultrasound, Point tracking, OpenCV, Neural network tracking, Tracking correction

## Abstract

Tracking points in ultrasound (US) videos can be especially useful to characterize tissues in motion. Tracking algorithms that analyze successive video frames, such as variations of Optical Flow and Lucas–Kanade (LK), exploit frame-to-frame temporal information to track regions of interest. In contrast, convolutional neural-network (CNN) models process each video frame independently of neighboring frames. In this paper, we show that frame-to-frame trackers accumulate error over time. We propose three interpolation-like methods to combat error accumulation and show that all three methods reduce tracking errors in frame-to-frame trackers. On the neural-network end, we show that a CNN-based tracker, DeepLabCut (DLC), outperforms all four frame-to-frame trackers when tracking tissues in motion. DLC is more accurate than the frame-to-frame trackers and less sensitive to variations in types of tissue movement. The only caveat found with DLC comes from its non-temporal tracking strategy, leading to jitter between consecutive frames. Overall, when tracking points in videos of moving tissue, we recommend using DLC when prioritizing accuracy and robustness across movements in videos, and using LK with the proposed error-correction methods for small movements when tracking jitter is unacceptable.

**Supplementary Information:**

The online version contains supplementary material available at 10.1186/s12938-023-01105-y.

## Introduction

### Tissue tracking and ultrasound

Visualizing deformations in skeletal tissues of the body during movement can be essential to understand relationships between body pose, muscle function, and movement. Understanding these features can also facilitate injury treatment and improve training, vital components of sports medicine and physical therapy. Ultrasound (US) is a comprehensive tool for this application, as it is one of the most used, least expensive, non-invasive forms of medical imaging available.

By far the most used form of US is brightness (B) mode, where transducer signals are converted into pixel brightness values [1]. When using US as a method of studying human motion, B-mode scanning is typically done before, during, or after a particular movement. In prior work, US images of the medial gastrocnemius muscle were collected every 24 h after delayed onset muscle soreness [2]. Changes in muscle area observed in 3 US images were correlated to power output and glycogen levels in cyclists [3]. In another study, US images of the biceps brachii muscle were acquired every 5 s throughout a series of contraction exercises [4]. These studies show that US images can be captured with varying time intervals, time instances, and probe positioning according the region of interest. They also describe how imaging tissue with US B-mode can be used to observe fatigue states, detect anatomical changes, assess injuries, and analyze other features of interest. We believe the diagnostic capabilities of US imaging can be expanded by tracking features of tissues in motion.

**Fig. 1:**
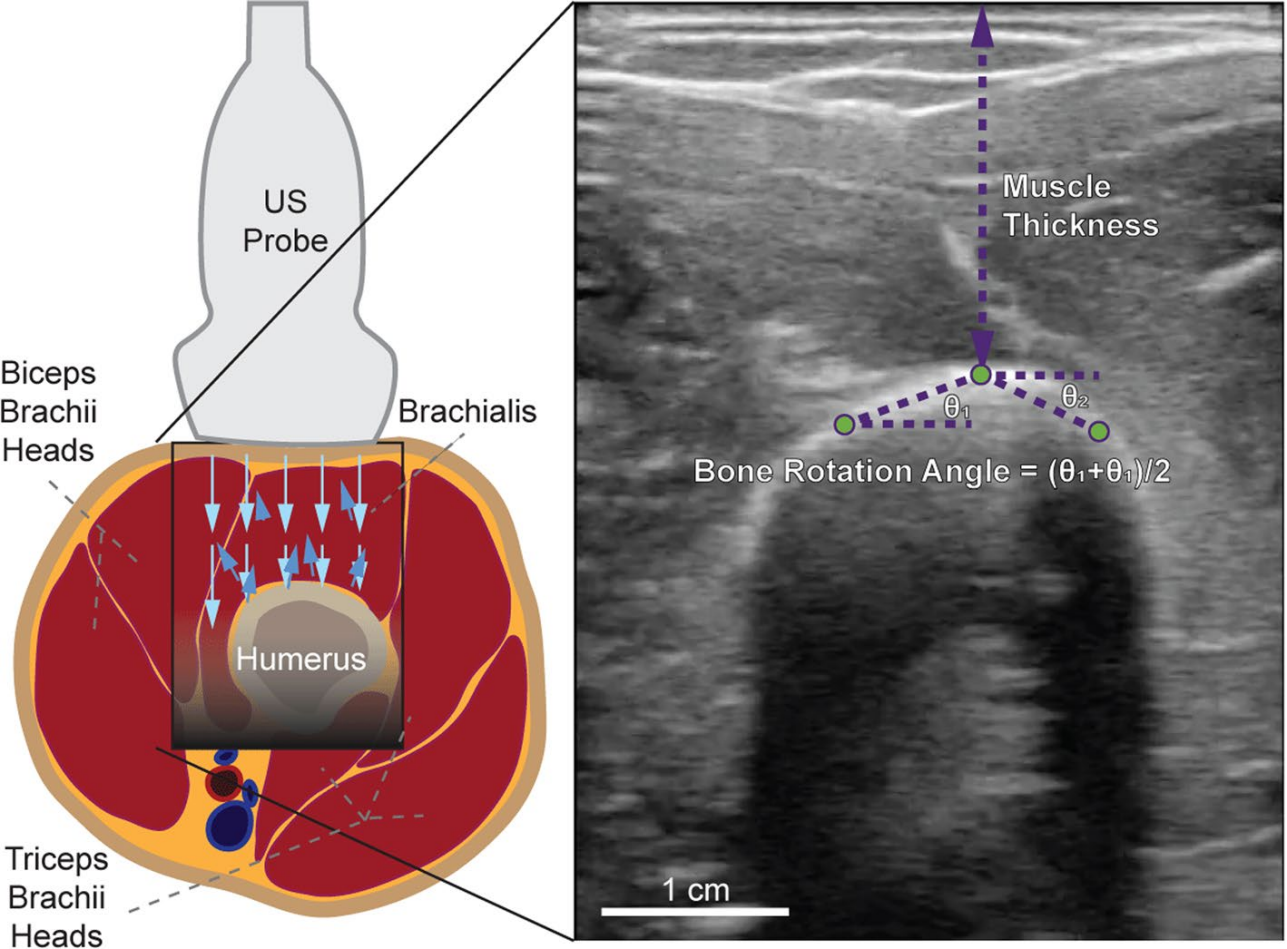
Ultrasound imaging setup. Ultrasound probe position on the upper arm for imaging the brachialis and the long head of the biceps brachii (left). A representative US image (right) showing how features such as muscle thickness and bone rotation can be estimated from tracking specific points of interest in ultrasound videos

When imaging skeletal tissue using US during movement, there are various common features of interest known as muscle architecture parameters that are correlated with muscle contractions and motion [5–8]. Features such as the pennation angle and fascicle length can only be observed when imaging the skeletal muscle longitudinally, and skeletal features such as bone rotation angle can only be observed when imaging the skeletal muscle transversally [9]. Other muscle architecture parameters include muscle thickness, cross-sectional area, and muscle displacement (Fig. 1).

Successes in US feature tracking have been observed in prior work, although registration and feature tracking in US is still a challenging and unsolved problem [10]. Feature segmentation algorithms such as the Rayleigh distribution [11], Curvelet transforms [12, 13], and curve fitting [14] have been used to distinguish regions of interest within the probe’s field of view. Speckle tracking was found to be as accurate as MRI observations when measuring ventricular torsion [15]. Optical Flow and block matching were combined to find sub-millimeter accuracy when measuring finger tendon motion [16]. Features such as pennation angle, thickness, and fascicle length of tissues were extracted from lower limb US video and used to estimate information about lower limb motion [9, 17]. These feature-tracking methods have all shown success, but have approached tracking on a case-by-case basis by developing custom algorithms and analysis pipelines, which can be time-consuming, computationally expensive, and challenging to generalize.

The ability to track increasingly smaller regions can establish a general methodology for tracking deformable features. Region and point-tracking algorithms such as Optical Flow [18, 19], block matching [20, 21], and template tracking [22] have been used to identify and track various tissues, tendons, and bones. Optical Flow uses pixel intensity (brightness) changes between two consecutive frames to determine pixel velocity and displacements [16] which can be helpful to track slow-moving features in any video. Block matching and template tracking can estimate the movement of a region of interest from one frame to another by maximizing the similarity between all regions in the next frame [16].

These methods often encounter various challenges due to the quality of US imaging. Optical Flow, for example, can fail with sudden movements, high speckle noise or fluctuating brightness. The issue of despeckling in particular has been addressed using filtering methods such as the Rayleigh Mixture Model [23], Daubechies complex wavelet transform [24], ripplet domain nonlinear filtering [25], and Laplacian pyramid-based nonlinear diffusion [26], which improve quality of US videos and tracking. Template tracking can encounter similar issues due to a short depth of view or poor image quality induced by the varying speed of sound characteristics of the imaged tissues. In addition, depending on the orientation of the probe and the body movement being observed, any point in the region of interest can inconsistently appear and disappear frame-to-frame. We encountered many of these challenges when tracking skeletal tissue features in the upper arm.

### Tracking algorithms

Here, we collected US videos of a transverse section of the upper arm, and labeled points in several frames of each video during various movements—walking, reaching, maximum voluntary contraction (MVC), and rest (see "Video collection"). Five tracking algorithms were used to track these points including four frame-to-frame trackers from the OpenCV library—Lucas–Kande (LK) [27], CSRT [28], MOSSE [29], and KCF [30]—as well as an open-source neural-network (NN) package—DeepLabCut (DLC) [31]. We will refer to the OpenCV trackers as “frame-to-frame” because they use information change between successive frames to track points.

Here, we show that point-tracking using frame-to-frame trackers are susceptible to error accumulation over time due to gradual drifting of the point being tracked. We also show that DLC-based tracking is not susceptible to this drift as CNN-based trackers do not make use of temporal information between frames. DLC-based tracking has the disadvantage of manual labeling and training a model to track points.

In this paper, we seek to improve frame-to-frame trackers so that they achieve DLC-level accuracy without the need to train a model. To achieve this, we propose three correction strategies—sigmoid tracking correction (STC), reverse tracking correction (RTC) and reversed sigmoid tracking correction (RSTC). These are heuristics-based methods that minimize drifting by interpolating the tracking result between manually labeled correction frames.

## Results

### Cumulative drifting occurs in all frame-to-frame trackers tested

**Fig. 2:**
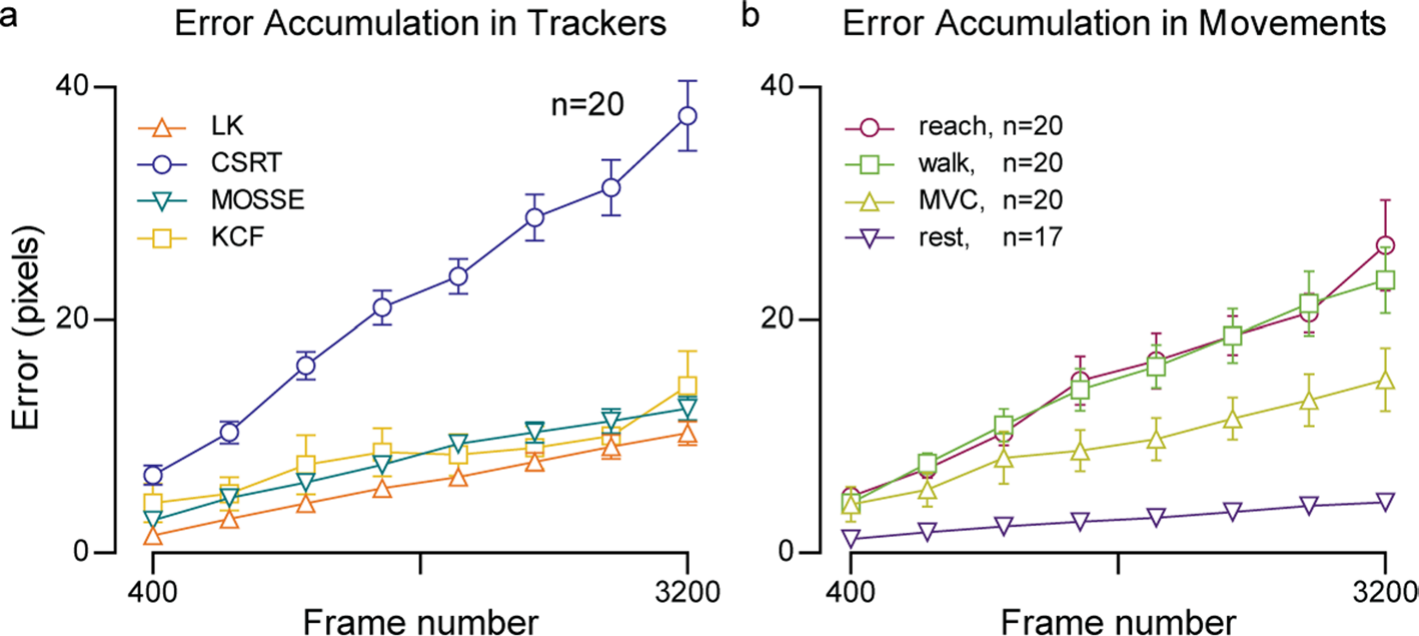
Error accumulation in frame-to-frame trackers. **a** Error in frame-to-frame trackers. The average error accumulation rate for the CSRT tracker, for example, is 0.0115 pixels/frame, or 0.88 μm/frame. **b** Error comparison across movements—walk, reach, maximum voluntary contraction (MVC), and rest (see “Video collection” for description). The error bars here show standard error of the mean (SEM)

To understand how cumulative drifting affected each frame-to-frame tracking algorithm, 3 distinct features were tracked starting at the first frame, tracking forward to *N* frames of a video, then tracking backward the same *N* frames. Since this process starts and ends in the same frame, the error was simply the average distance between the point locations in the first and last frames. Figure 2a shows average error per tracker type across all movements, and Fig. 2b shows average error per movement type across all trackers. Accuracy (error) values are calculated as the average distance between the labeled points and the tracked points in pixels. In this study, 13 pixels correspond to 1 mm. Raw data presented in all the results can be found in Additional file 2.

From Fig. 2a, it is clear that the CSRT tracker experienced the most drifting (${\overline{x} }_{\mathrm{CSRT}}=36.8$ pixels over 3200 frames) relative to the other trackers (${\overline{x} }_{\mathrm{KCF}}=14.1$ pixels for KCF, ${\overline{x} }_{\mathrm{MOSSE}}=12.2$ pixels, ${\overline{x} }_{\mathrm{LK}}=10.1$ pixels over 3200 frames). In addition, Fig. 2b shows that large tissue movements during reaching and high-impact activities such as walking resulted in more drifting by the trackers (${\overline{x} }_{\mathrm{walk}}=23.4$ pixels, ${\overline{x} }_{\mathrm{reach}}=26.4$ pixels over 3200 frames). Despite some trackers and movements resulting in lower cumulative errors than others, Fig. 2 shows that drifting is a challenge present in frame-to-frame trackers across the board.

### Approaching drifting correction using three novel methods

**Fig. 3:**
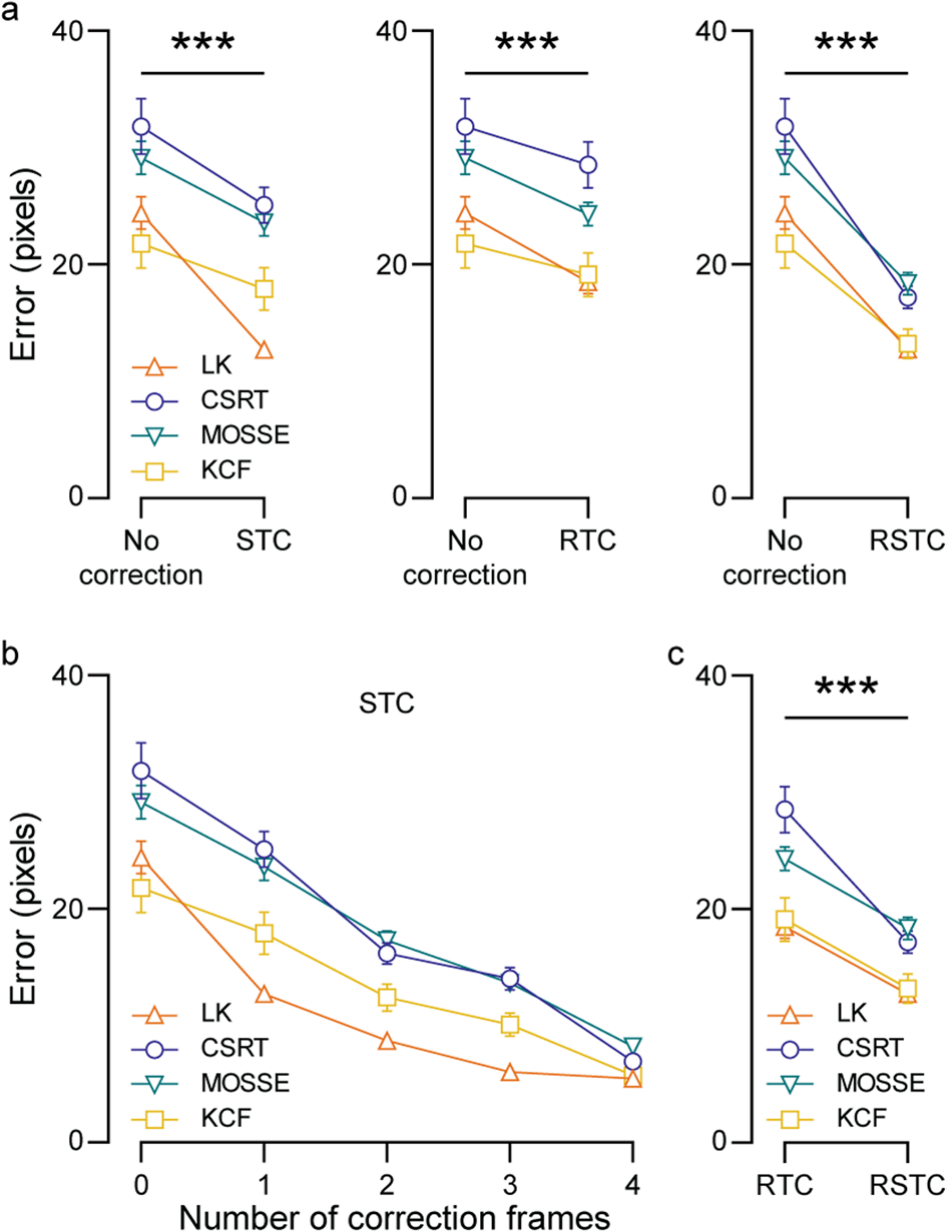
Accuracy improvement following drifting correction methods. **a** Tracking accuracy significantly improves upon STC (left), RTC (middle), and RSTC (right) correction methods. **b** Effect of increasing the number of correction frames on tracking accuracy with STC correction. **c** Comparison of tracking accuracy between RTC, and RSTC corrections. Error bars refer to SEM across the 20 subjects (*** $p<0.001$)

Sigmoid tracking correction (STC) is our first proposed method to minimize drifting, and requires at least two frames with points labeled—one initial frame for the trackers to start on and at least one “correction” frame to reset the tracker. We ran each tracker from the initial labeled frame to the first correction frame, reset the points, and similarly continued the process for an increasing number of correction frames all equally spaced apart. If more correction frames were used, the space between two correction frames decreased accordingly. After tracking was complete, we added a sigmoid to smooth out the tracking path, as described in “Sigmoid tracking correction”. Figure 3b summarizes these results.

Next, to use reverse tracking correction (RTC) and reverse sigmoid tracking correction (RSTC), we ran each tracker forward and backward in time. For RTC, the forward and backward tracked paths were averaged (“Reversed tracking correction (RTC)”). For RSTC, the contribution from the forward path near the initial labeled frame was increased, and the contribution from the backward path near the last labeled frame was increased (“Reversed sigmoid tracking correction (RSTC)”). We then compared the errors when no drifting correction was used, when RTC was used, and when RSTC was used (Fig. 3).

As expected, using more correction frames with STC reduced the average error, with significant improvements with just 1 correction frame (Fig. 3a, left panel; paired *t*-test, $n=80, t=12.21$, $p<0.0001$). STC provides an efficient way to reduce tracking error using labeled frames to combat drifting of the traced feature (Fig. 3b). However, it is important to note that error is measured at these frames, so when more correction frames are used, more errors equal to 0 will contribute to the average shown in the figure.

RTC and RSTC correction methods with just two labeled frames significantly decreased tracking error (Fig. 3a, middle and right panels; RTC—paired *t*-test, $n=80, t=8.509$, $p<0.0001$; RSTC—paired *t*-test, $n=80, t=16.47$, $p<0.0001$), and using RSTC also significantly decreased error when compared to using RTC (Fig. 3c; paired *t*-test, $n=80, t=14.66$, $p<0.0001$) among all trackers. LK and KCF consistently resulted in the lower error when compared to CSRT and MOSSE.

The key takeaway for Fig. 3 is that the STC, RTC, and RSTC methods significantly improve the accuracy of frame-to-frame trackers (Fig. 3a), RSTC correction is superior to RTC (Fig. 3c), and LK is the most accurate among the LK, CSRT, MOSSE and KCF trackers tested (Figs. 2, 3). Therefore, for further analysis, we combine the STC and RSTC correction methods with LK tracker (Fig. 5).

### DLC proves accurate and efficient post-training

**Fig. 4:**
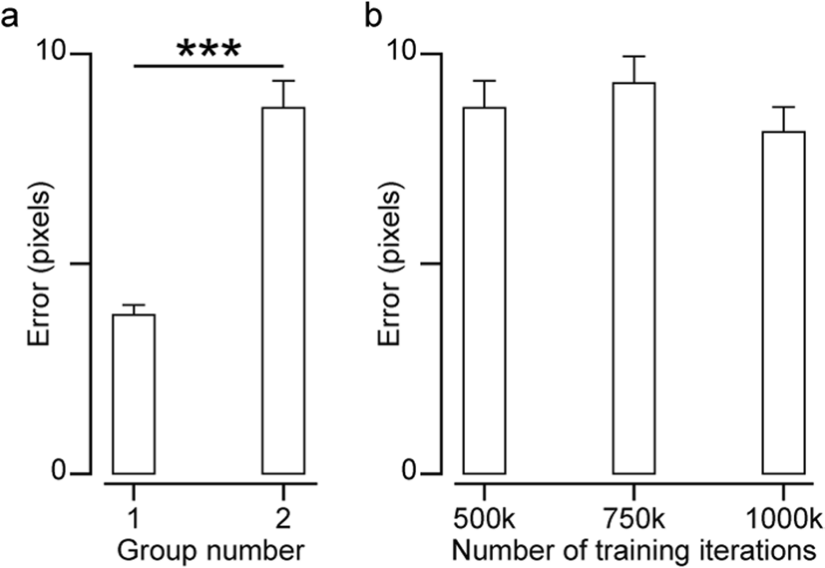
Tracking accuracy for DLC. **a** Tracking accuracy when using one DLC model per subject (Group 1) versus one model per group of subjects (Group 2). **b** Tracking errors when increasing the number of training iterations for DLC model across subjects (Group 2). The datapoints for Group 1 come from the average errors in 20 subjects, 6 videos each. The datapoints for Group 2 come from the average errors over 20 randomized video groups among 6 subjects. Error bars represent SEM (*** $p<0.001$)

Up to now, we analyzed the frame-to-frame trackers among one group of subject videos (Group 1) in which each subject’s US videos were labeled with a unique template. Next, we used DLC to train individual models for each subject in Group 1 with the same labeled frames used as ground truth to test frame-to-frame trackers. We found the average test error to be 3.8 ± 0.2 pixels (Fig. 4a), lower than that obtained with any frame-to-frame trackers using drifting correction (Fig. 3).

It is likely these models performed this well because we trained one model per subject, meaning each model only learned the morphological characteristics of one bone-muscle group. To test the effectiveness of a generalized DLC model across multiple subjects, we used videos from a second group of subjects (Group 2) and created a general template frame with three labeled points along the edge of the humerus (Fig. 9). After labeling all videos in Group 2, we trained three models using 80% of the videos—one using 500,000 training iterations, one using 750,000, and one using 1,000,000—to understand the sensitivity of model accuracy to the number of training iterations. The remaining 20% of videos were randomly placed into 20 groups of 6 videos each, and the three models were applied to these groups. For each model (500 k, 750 k, 1000 k), the errors of all 20 groups were found, and their accuracy is shown in Fig. 4b.

As expected, the error in the 500 k-iteration Group 2 model was significantly greater than that of the 500 K-iteration Group 1 models (unpaired *t*-test, $df=38, t=7.18$, $p\ll 0.001$), as the Group 2 model learned the characteristics of multiple bone-muscle groups to track the given points. The average errors in Group 2 were not significantly different among the models with increasing training iterations (500 k to 750 k to 1000 k, one-way ANOVA, $df=35, F=0.26, p=0.77$), showing that DLC is robust and consistent across the number of training iterations used.

If DLC is chosen to track features in these types of US videos, we recommend using 500 k training iterations as it has comparable accuracy to higher iteration models while being less costly to train.

### Combining corrections to frame-to-frame methods to approach DLC accuracy

Tracking error when using DLC is lower than uncorrected frame-to-frame tracking (Figs. 3,4). Here, we seek to identify a correction strategy that improves the accuracy of frame-to-frame trackers to approach that of DLC tracking.

We knew from Figs. 2a that the average LK drifting error of ~ 0.3 μm per frame. DLC’s Group 1 models had an average error of ~ 300 μm, implying that LK is more accurate than DLC for tracking points in videos less than about 1000 frames. With this in mind, we combined these methods using every 200th frame tracked by DLC as a pseudo-labeled frame, tracking forwards and backwards from each of these frames using LK, and applying RSTC to reduce drifting effects (LK + RSTC + DLC).

**Fig. 5:**
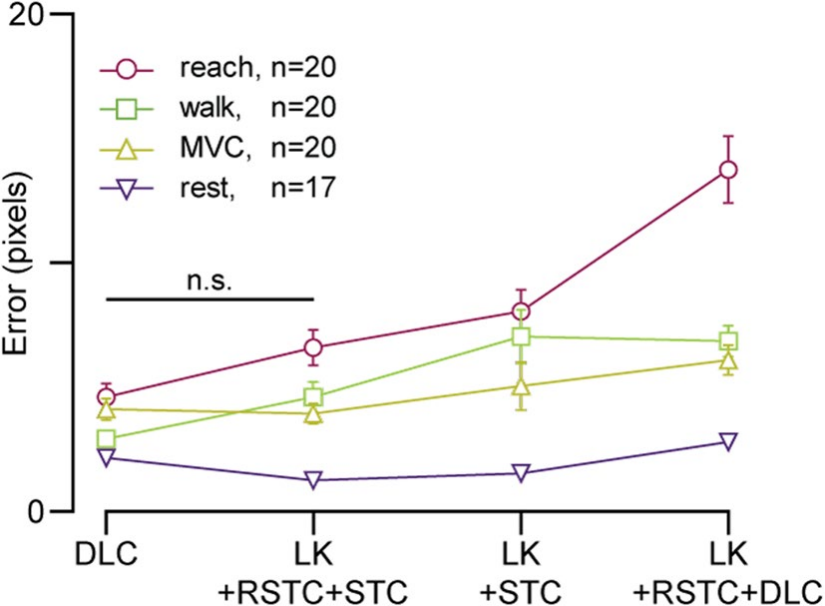
Errors when combining drifting correction methods: accuracy of methods combining LK and DLC tracking algorithms with proposed STC and RSTC methods of drifting correction. Error bars represent SEM

We also observed in Fig. 3a that using STC with 4 correction frames resulted in a low average error of 6.6 pixels among all trackers, but the contribution to that average from LK was an error of 5.5 pixels (LK + STC). In an attempt to decrease this, we combined STC and RSTC by tracking from each of the 4 correction frames forwards and backwards with LK, and applying RSTC to these paths (LK + RSTC + STC). Finally, we grouped the errors to compare each method’s sensitivity to movement, shown in Fig. 5.

This figure presents one of the main advantages of using DLC—robustness. The range of errors when using DLC was smaller than any other method here, showing it was less sensitive to large or fast tissue movements when tracking. However, using LK + RSTC + STC resulted in an error not significantly different than that obtained by DLC across movements (paired *t*-test, $n =4$, $t=0.918$, $p=0.427$), with DLC outperforming LK + RSTC + STC for quick and/or large movements in tissue (walk—paired *t*-test, $n =20$, $t=2.909$, $p=0.009$; reach—paired *t*-test, $n =20$, $t=2.688$, $p=0.0145$), and the tables turning for small and/or slow movements (MVC—paired *t*-test, $n =21$, $t=0.360$, $p=0.723$; rest—paired *t*-test, $n =17$, $t=9.418$, $p<0.0001$).

### DLC tracking has more jitter than recommended combined frame-to-frame method

**Fig. 6:**
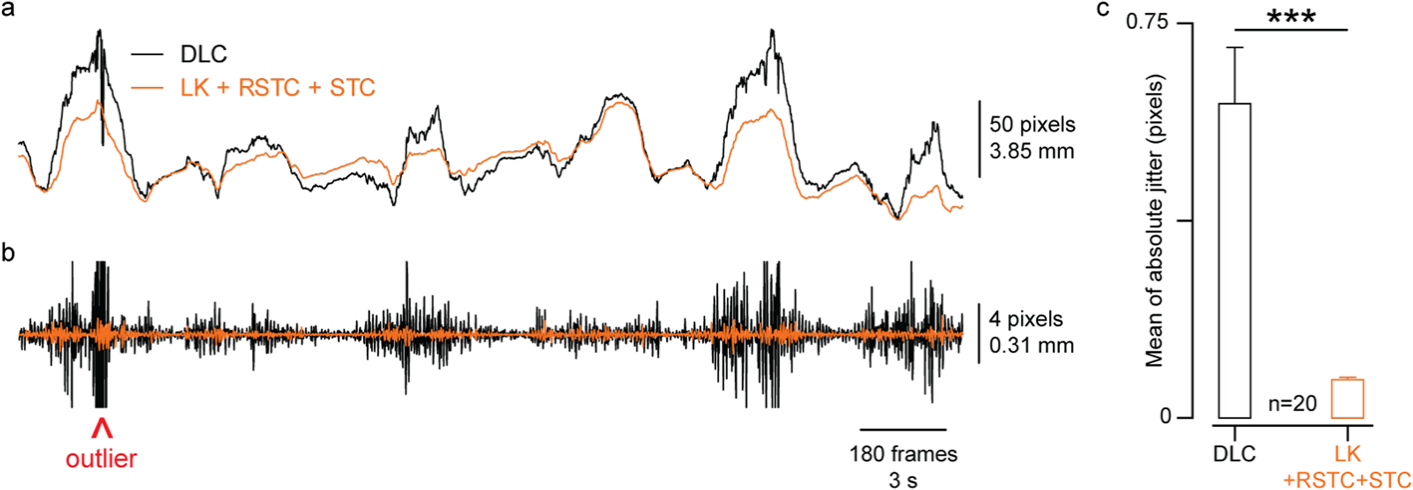
Comparing tracking jitter in DLC and LK with correction. **a** A representative example of a tracked point’s *x*-coordinate with DLC tracking in black and LK + RSTC + STC tracking in orange. **b** High-pass filtered (> 15 Hz) signals from (**a**) to contrast the amount of jitter with these two approaches. Outlier from DLC tracking shown with red arrow. **c** Group data contrasting the average amount of absolute jitter. Error bars represent SEM across 20 subjects (*** *p* < 0.0001)

When tracking these videos, we observed that the points jittered more when using DLC than using LK with RSTC and STC. A few jitters seen were due to tracking outliers (discussed in “Tracking”) which can be mostly eliminated by adding more labeled frames. The more consistent jitter likely comes from the non-temporal tracking of CNN trackers that estimate the point location(s) at every frame independently of the frames right before or after it. LK, on the other hand, exploits spatial and temporal information by searching a point’s location in the prior frame to determine its movement, leading to a smoother path. Figure 6 gives an example of this difference.

This figure presents the primary caveat to using DLC. Though accurate and robust to video variability, DLC tracking jitters much more strongly than LK, making it an unattractive choice to track small tissue movements. To contrast jitter in LK and DLC, we quantified jitter as the average of the absolute value of the high-passed signal and found the jitter in tracking with LK + RSTC + STC to be significantly lower than jitter in tracking with DLC (Fig. 6c, paired *t*-test, $n=20,$ $t=4.952$, $p<0.0001$).

## Discussion

### Tracking

Among all frame-to-trackers analyzed, LK had the least error due to drifting, which is perhaps the reason LK is used often to analyze US videos [22, 32, 33]. For analyzing small changes in imaged tissues, for example during the rest condition (“Video collection”), results from LK were visually more pleasing and preferred to DLC results due to the lack of jitter (Fig. 6).

Combining DLC and LK (LK + RSTC + DLC) increased error compared to using DLC alone, going against our initial hypothesis that using DLC tracking to aid LK would decrease error. Further inspection showed that when insufficient labeled data is given to DLC, it is unable to confidently and fully track the points, resulting in several “outlier” frames. DLC has a feature to relabel these outlier frames, but we chose not to use it to maintain the quantity of labeled information across trackers. Because of this, DLC outliers created discontinuous jumps in tracking from one frame to another, often skewing LK to track away from the desired feature, and increasing the error as a result. Further research is required to combine diverse tracking algorithms such as Optical Flow and CNNs in a meaningful manner to achieve accuracies better than either of the methods in isolation.

Since DLC tracking does not use temporal information, we reasoned that post-processing corrections that use temporal information, such as Kalman filtering or low-pass filtering can potentially reduce jitter in tracking and improve tracking results. Counter-intuitively, even though these methods reduced jitter, the tracking error did not decrease, and in some cases, such as walking motion, the tracking error instead increased (Additional file 1: Sect. 1.4 and Fig. S4). Further research is required to identify appropriate post-processing algorithms and successfully combine them with point-tracking algorithms.

### Labeling

A caveat that might limit the accuracy of all trackers including DLC is labeling. Manually labeling US videos sometimes required rough estimates of a feature’s position, especially when tissue moved out of the imaging field of view during large arm movements. These estimates could have biased the error values, especially for DLC which already had a very low error.

The sparsity of the frames chosen to be labeled might have also caused errors in accuracy. As later described in “Methods”, 8 equally distributed frames in each video were labeled. For short ~1-min-long videos, the gaps between each labeled frame lasted ~ 7 s (400–500 frames), while the gaps for longer ~ 3-min videos lasted ~ 20 s (1200 frames). The errors were only measured at these labeled frames, so no information about the quality of tracking during these gaps was measured, potentially missing tracking failures. We hope that by averaging errors from videos of various movements and various durations, the error variations were averaged out.

Manually labeling correction frames is time-intensive, and the optimal number of correction frames for LK tracker can be chosen based on the movement type and error tolerance based on the results presented in Fig. 2b. For example, the correction frames would be spaced at shorter intervals for reaching and walking compared to maximum voluntary contraction movement. When training one DLC model per subject, labeling a total of 24 frames per model was sufficient to have an average test error below 4 pixels. The number of labeled frames depends on training one model per probe position for DLC, whereas it depends on the amount of data collected for LK with RSTC and STC.

DLC had an average error lower than 4 pixels in Group 1 after labeling 24 frames per probe placement (in this case one probe placement per subject). The total number of labeled frames for the per-subject model approach was 1440 (20 subjects × 3 points per frame × 24 frames per subject), rendering it potentially infeasible for quick analysis. Current DLC models are manually intensive for creating training data, time consuming to train, and computationally intensive. To optimize tracking accuracy without training a model, we recommend using LK with RSTC and STC corrections based on our findings.

### Hardware

A consideration for any study using US probes is their potential impact to deform elastic tissues during an experiment. US requires constant contact with the area of interest, often paired with pressure to maintain the probe at a unique location. In this application, the probe was strapped pointing perpendicularly into the arm with self-adhesive tape tightly wrapping around it. This tape was flexible and allowed the upper arm muscles to contract and relax, but constraining the arm in this manner might have affected how the skeletal tissue took shape during fast movements.

The b-mode ultrasound videos captured in this study are from a linear probe providing a cross-sectional view into a portion of the upper arm. The approaches proposed in this work are only meaningful for features that stay in the imaging plane, which can vary with the type of motion. This is a significant challenge for characterizing tissues in motion, and can be addressed through hardware innovations that allow for volumetric imaging. The proposed methods would then have to be adapted and evaluated for point tracking in 3D space.

### Applications

Ultrasound technology can be used to evaluate muscle function non-invasively during motion, which could illuminate the role of elastic tissues in movement, preventing injury, and promoting recovery. Algorithmic improvements and innovations are essential for extracting quantitative information from ultrasound data and precise evaluation of muscle function. The strategy of point tracking can be applied to track changes in features such as muscle cross-sectional area, muscle thickness, pennation angle, and bone-muscle interaction during motion. Even though point tracking is the focus of this work, other strategies such as image segmentation can also be useful for quantitative muscle function evaluation using ultrasound.

Improving the precision of point tracking in b-mode ultrasound videos can potentially aid in understanding and managing tremors in healthy individuals and patients with movement disorders. If a much larger dataset were to be labeled by expert sonographers, better neural-network models that generalize well across regions of the body and across subjects can be trained to quantify tissue movement with a much lower tracking error than shown in Fig. 4a for Group 2.

## Conclusion

US is an inexpensive and non-invasive tool to visualize skeletal muscle during movement, but tracking features in B-mode US using Optical Flow-based trackers presents many issues including point drifting. In this paper, we show that drifting is a recurring challenge when tracking in US, and propose various correction algorithms to minimize this phenomenon. We show that using Lucas–Kanade (LK) for this application results in the lowest tracking error of the frame-to-frame trackers that we explored, and applying these correction algorithms improves accuracy.

We show that DeepLabCut (DLC) outperforms all non-modified frame-to-frame trackers, not only being minimally sensitive to the type of movement but also generally resulting in lower error. The only caveat against DLC is the jitter that can sometimes result in noisy tracking.

In conclusion, we recommend DLC for point tracking in ultrasound videos due to its accuracy and robustness, except for tracking small movements in tissues, where we recommend LK in combination with the proposed correction algorithms.

## Methods

In this section, we describe the experimental setup for collecting US videos, our proposed enhancements to point tracking, and methods to compare the results of various trackers.

### Video collection

26 healthy subjects (13 female, 13 male) aged 27.9 ± 7.9 years participated in a study held at the MIT.nano Immersion Lab. All subjects provided written informed consent according to MIT’s Committee on the Use of Humans as Experimental Subjects (COUHES). Data were collected using a Cephasonics Cicada Ultrasound system (Cephasonics Ultrasound Solutions, San Jose, California, USA) and a 7.5 MHz 128 channel linear ultrasound probe. The probe was attached to the brachialis of a randomly selected arm in order to capture a transverse view of its internal morphology—this arm will be referred to as the “US arm”. The probe captured B-mode images at ~60 frames-per-second. Figure 1 shows an example view.

**Fig. 7:**
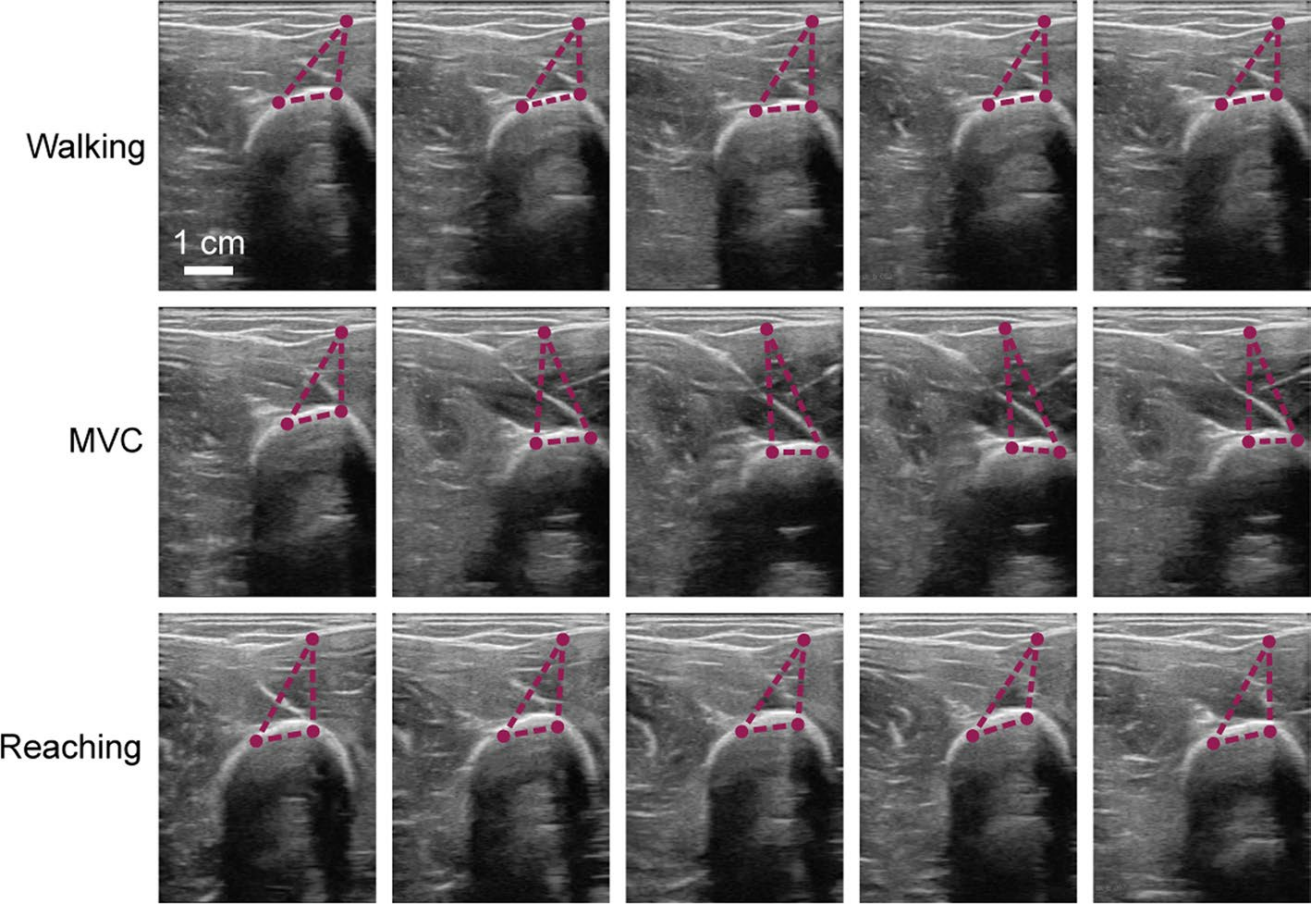
Sequence of US frames throughout various movements: highlighting how various types of movements during walking affect the morphology of the skeletal muscle in the upper arm

During the experiment, each subject performed a sequence of movements meant to invoke various morphological changes. The first was walking, invoking quick tissue movements from the impact of each step. The second was maximum voluntary contraction, invoking quick but small tissue movements due to muscular co-contraction. The third was reaching, invoking slow progressions between stretched and unstretched muscles. The last was resting, invoking small changes in the morphology. Figure 7 displays a sample sequence of frames for some of these movements, highlighting how various points in tissues shift and twist as the movement occurs.

We collected between 20 and 30 videos per subject each lasting between 1 and 3 min depending on the movement, resulting in a range of 3600 to 13,000 frames per video, with the analyzed videos averaging about 6400 frames long. Data were derived from 20 subjects for all analyses, except for when training a cross-subject DLC model (Fig. 4), where data were derived from another subject group. One video was chosen per movement per subject, with the exception of the MVC movement, where 2 videos were chosen from one of the subjects, and the average error across these two videos was used for that subject. For the rest condition, one video was chosen per subject from 17 subjects.

### Human labeling

**Fig. 8:**
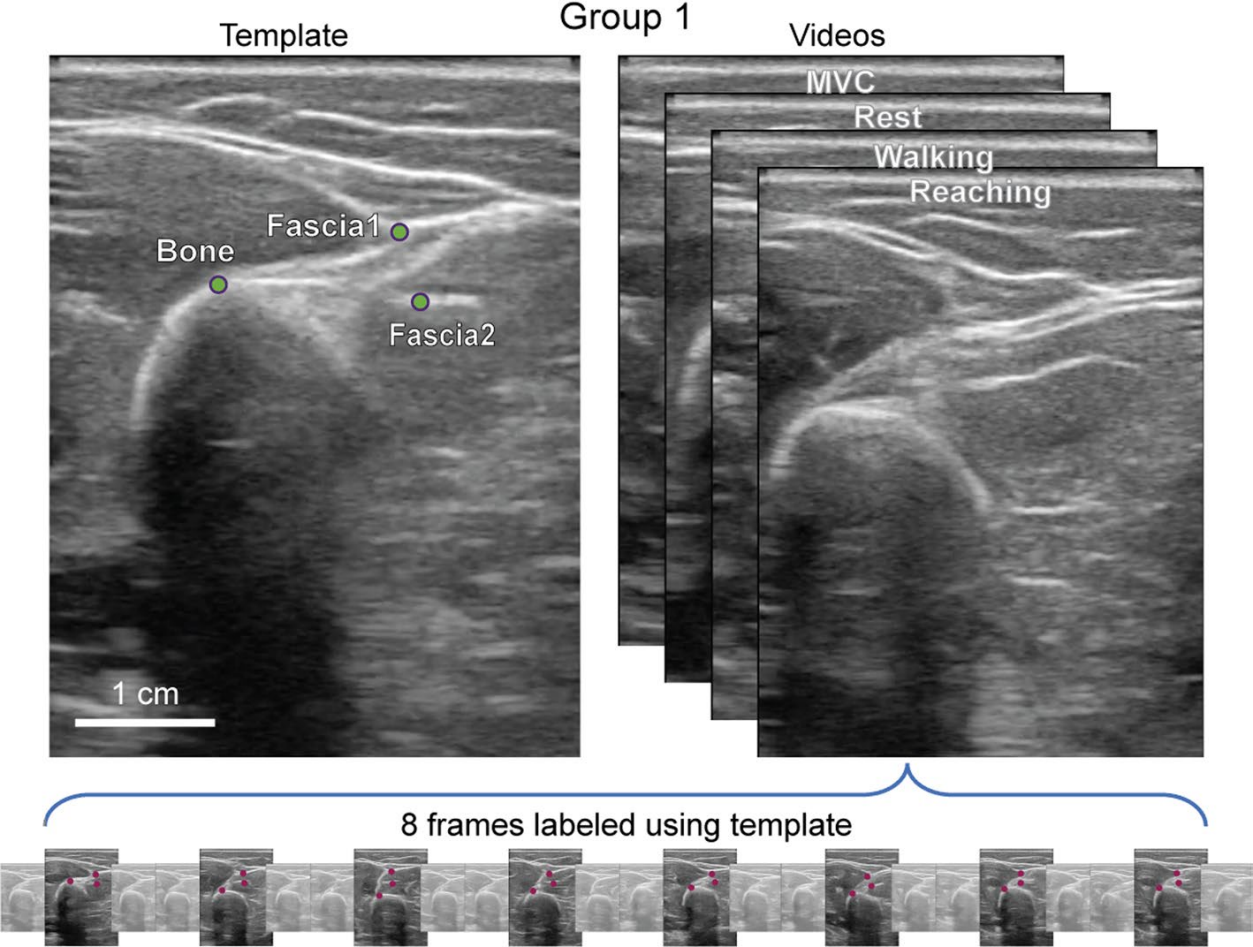
Labeling process for Group 1: the tracking template for an example subject is shown at the top with green labeled points. In the bottom panel, labeled points from 8 equally spaced frames across the video are shown in red

**Fig. 9:**
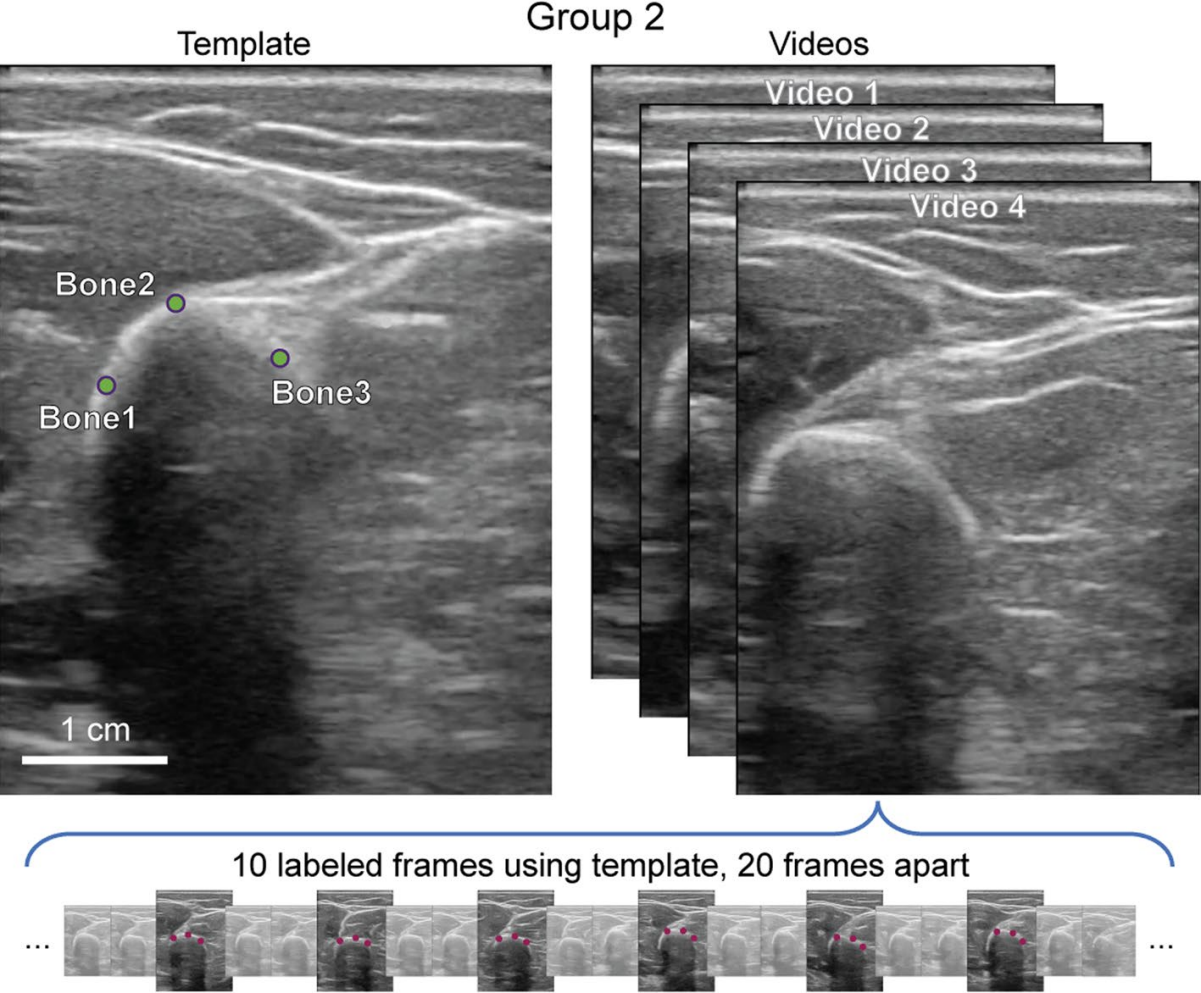
Labeling process for Group 2: the left panel shows an example template for the second video labeling process. The right two frames show approximate methods for obtaining relative bone rotations and muscle thickness

Human labeling was used as ground truth to evaluate the performance of tracking algorithms. A portion of this data was also used to train the DLC-based tracker. Subjects were divided into groups, and data from each group was labeled differently (Figs. 8 and 9. Features unique to an individual were chosen in Group 1, and features common across individuals were chosen in Group 2. For DLC tracking, one model was trained per subject in Group 1, and one model was trained across all subjects in Group 2.

Features chosen in Group 1 were prominent landmarks on the bone and fascia specific to the probe positioning that would not go off-frame in any of the videos. A template was first created per subject by marking these features on one frame (Figs. 8 and 9, left panel). Then, eight frames equally separated in time were chosen for each movement type (walking, maximum voluntary contraction, rest, and reaching) from one randomly selected video and labeled using the template as a reference. Figure 8 illustrates this process.

With this process, each subject had a total of 48 labeled frames with 3 labeled points, or 144 total labeled points used by tracking algorithms and used to assess each algorithm’s tracking accuracy.

The ultimate goal of this process was to find the most accurate tracking algorithm independent of the type of feature being tracked. Having each subject’s template highlight different features prevented tracking to be generalized for this purpose. Therefore, we collected a second group of videos from a six subjects, and three points along the edge of the bone were labeled as shown in Fig. 9. For this group, each subject had between 13 and 20 US videos. 10 frames that were 0.33 s (20 frames) apart were labeled. Choosing a shorter gap between all labeled frames of this batch of subjects allowed for a more precise manual labeling process, especially because only small and clear movements would occur from one labeled frame to another.

The more points that are labeled along the edge of the bone or other tissues, the more accurate these feature approximations can be.

### Frame-to-frame tracking algorithms

#### OpenCV

The first set of algorithms used are frame-to-frame trackers within the OpenCV library. These algorithms were analyzed extensively on various types of videos [33–36], and were used to track pedestrians in a crowd, vehicles, or faces. Although many image-processing algorithms have been developed to tackle specific tracking challenges in US video (liver tracking [37], surgical instruments [38]), little work has been conducted comparing the accuracy of OpenCV algorithms. Imaging the upper limbs transversally can specifically display detailed cross-sections muscle thicknesses, bone rotation, and fascia distributions, but it has yet to be thoroughly analyzed using OpenCV.

As of writing this manuscript, OpenCV has 8 major frame-to-frame trackers including CSRT (Channel and Spatial Reliability Tracker) [28], MOSSE (Minimum Output Sum of Squared Error) [29], KCF (Kernelized Correlations Filter) [30], BOOSTING [39], MIL (Multiple Instance Learning) [40], MedianFlow [41], TLD (Tracking Learning Detection) [42], and GOTURN (Generic Object Tracking Using Regression Network) [43]. Analysis of only the CSRT, KCF, and MOSSE trackers was performed due to promising prior results from non-US video tracking [34] and pilot analyses.

Most of the issues that these trackers might encounter arise from feature obstruction, fast feature movement between frames, feature movement away from the field of view, and tracker drifting. Imaging the arm transversely made it possible for similar issues to be encountered since tissue features could travel out of the plane; however, various aspects of tracking in this US application actually lessen these burdens. First, tissues remain close and in the same general orientation relative to each other. Second, features that exit the plane of view during one movement return to the plane during the reversal of that movement. And third, once the US probe is attached to a specific location, the average size of morphological features do not vary greatly. These benefits when tracking US videos are not often found in the other common pedestrian or vehicle applications.

In order to begin tracking, all OpenCV algorithms require a bounding box that acts like a template for some of these trackers to capture frame-to-frame movement of image features. A 100 × 100 pixel square was used as the initial bounding box size, but smaller square sizes were also used for comparing tracker accuracy and speed. After each update to the bounding box, the center point of the new box was found, and the new box’s size was reset to its initial dimensions to avoid the potential distorting of box dimensions over time.

#### Lucas–Kanade (LK)

The Lucas–Kanade (LK) method [27] has been one of the most widely used computer vision techniques for tracking, motion estimation, face decoding, etc. [44] for the last 30 years. From studying the gastrocnemius medialis tendon in [22] to tracking the internal jugular vein in [32] to tracking the carotid artery in [33], Optical Flow algorithms like LK are observed to be generally accurate when compared visually or quantitatively to ground truth. LK uses a template image $T(x)$, usually a small subsection of a video frame, and aligns it to an image of interest $I(x)$, usually the video’s next frame. A parameterized warp matrix $W(x;p)$ takes some pixel of the template $T(x)$ and maps it to the coordinate frame in the image $I$. This matrix allows the algorithm to check how various features are not only moving, but also distorting throughout the video. The algorithm is described in [44], but a brief summary is presented here.

The goal of the algorithm was originally to find where the template image likely moved to in the next frame, regardless of how the template might have distorted between frames. Mathematically, this refers to minimizing the sum of squared errors between the template $T(x)$ and the image $I$ warped back onto the template’s coordinate frame:

1 $$\min \sum\nolimits_{x} {\left[ {I(W(x;p)) - T(x)} \right]}^{2}$$

Some $\Delta p$ is computed using the partial derivatives of the gradient of $I$ evaluated at $W(x;p)$. This value is updated until the norm of the vector is below some threshold.

while $$||\Delta p||<\epsilon$$:

compute $$\Delta p$$

$$p+=\Delta p$$

The OpenCV adaptation of the algorithm simplifies this method by simply minimizing the sum of squared errors between a template $T(x)$ and the next image in a video $I$, to avoid considering warping effects. Under the assumption that the pixels in the template continue to have the same size, shape, and intensity, one of these pixel’s change as follows:

2 $$I(x,y,t)=I(x+\mathrm{d}x,y+\mathrm{d}y,t+\mathrm{d}t)$$

which can be simplified via Taylor-series approximation to:

3 $$\frac{\delta f}{\delta x}\frac{\delta x}{\delta t}+\frac{\delta f}{\delta y}\frac{\delta y}{\delta t}+\frac{\delta f}{\delta t}=0$$

4 $${f}_{x}u+{f}_{y}v+{f}_{t}=0$$

Expanding this to all $n\times n$ pixels in the template, the problem expands to solving ${n}^{2}$ equations with 2 unknowns, which can be estimated with a least-squares to get the solution:

5 $$\left[\begin{array}{c}u\\ v\end{array}\right]={\left[\begin{array}{cc}{\sum }_{i}{{f}_{{x}_{i}}}^{2}& {\sum }_{i}{f}_{{x}_{i}}{f}_{{y}_{i}}\\ {\sum }_{i}{f}_{{x}_{i}}{f}_{{y}_{i}}& {\sum }_{i}{{f}_{{y}_{i}}}^{2}\end{array}\right]}^{-1}\left[\begin{array}{c}-{\sum }_{i}{f}_{{x}_{i}}{f}_{{t}_{i}}\\ -{\sum }_{i}{{f}_{y}}_{i}{f}_{{t}_{i}}\end{array}\right]$$

This solution provides the corresponding velocity vector of a particular pixel at that time.

#### Anti-speckling

Initial visual inspection of these algorithms revealed that drifting occurred consistently, especially in longer videos with more tissue and bone movement. To partly tackle this issue, the wavelet denoising algorithm from Python’s Scikit library was used prior to tracking, as it was shown to increase the Peak Signal to Noise Ratio (PSNR) of each frame. Tracking visually improved after this process.

### Drifting correction algorithms

#### Sigmoid tracking correction (STC)

In general, the issue of cumulative drifting in the frame-to-frame algorithms described above was decreased significantly with anti-speckling techniques when analyzing videos with a low-movement activity, such as when resting. This unfortunately did not fully eliminate the problem for videos with a high-movement activity. In an attempt to minimize the effect of drifting using labeled data, an algorithm that corrects drifting motion over time is proposed.

The movements performed by the subjects were both slow and repetitive which made the drifting patterns of the bounding boxes somewhat predictable. If drifting did occur during tracking, it often had a slow and steady progression away from the feature of interest. For this reason, a method called sigmoid tracking correction (STC) is proposed.

The idea behind STC is relatively simple—if a point being tracked drifts away from the desired feature, and you have labeled data at some initial and end frames, a smooth function can be added to connect the labeled points while maintaining most of the higher-frequency movements during tracking. More explicitly, say a point’s coordinate at frame *i* is ${x}_{i}$ and the point’s coordinate at the next labeled frame i + *T* is ${x}_{i+T}$

When the point is tracked from frame *i* to frame i + *T*, it drifts to some: ${x}_{i+T}+d$

**Fig. 10:**
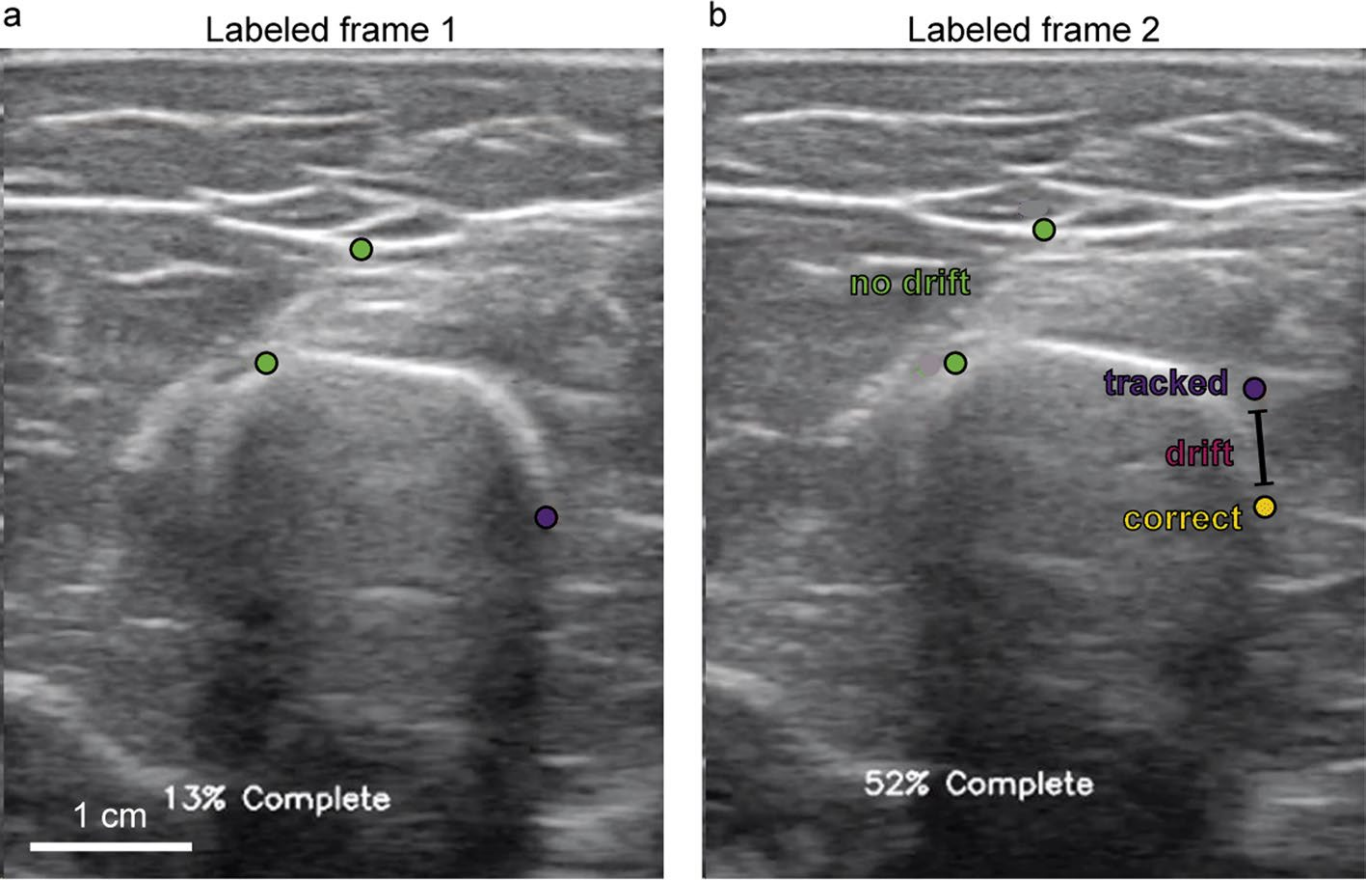
Tracking drifting example. **a** shows the video at a labeled frame, and **b** shows the video at the next labeled frame, with the yellow point representing where the purple point on (**a**) should have been tracked to

An example of this drifting is shown in Fig. 10 below. As tracking occurs from some initial Labeled frame 1 to the next Labeled frame 2, the original yellow point in Fig. 10a drifts away from the desired (purple) point on Fig. 10b. The drift value $d$ is, therefore, just the distance between the yellow and purple points on Labeled frame 2.

To correct the point’s drift, a sigmoid function is added to the frame’s tracked coordinates between some initial and following labeled frame. Sigmoids have the form:

6 $$S(x)= \frac{1}{1+{e}^{-x}}$$

which are smooth functions that approach 0 and + 1 as $x$ approaches − ∞ and + ∞, respectively, with a maximum slope at $x=0$. The function can be parameterized to the form:

7 $$S(x)= \frac{A}{1+{e}^{-B(x-C)}}+D$$

and tuned to shift this function accordingly. The value of $A$ scales the height of the function as $x$ approaches + ∞, the value of $B$ determines the maximum steepness of the function ($\mathrm{slope}=\frac{AB}{4}$) at its midpoint, the value of $C$ determines the horizontal shift of the function, and $D$ determines the vertical shift of the function. This function only approaches its asymptotes, so the drifting path error can also only be corrected up to a small tolerance $\epsilon$ times the error $d$. An $\epsilon$ of 0.01 is chosen in this case to reach a 1% error. Therefore, the new coordinate after summing the appropriate sigmoid function at the initial labeled frame $j=i$ should be

$${x}_{\mathrm{new},j}={x}_{j}+\epsilon d$$

and the new coordinate right before the next labeled frame $j=i+T-1$ should be

$${x}_{\mathrm{new},j}={x}_{j}+(1-\epsilon )d$$

Knowing the coordinate should approach ${x}_{j}+\epsilon d$ instead of 0 at − ∞, and ${x}_{j}+(1-\epsilon )d$ instead of 1 at + ∞, it can be concluded that the vertical shift to the sigmoid function should be $D={x}_{i}$. The horizontal shift to the function is determined by the location of the midpoint which is $C=0$ for the standard sigmoid. For this application, the midpoint between the initial and end frames (and therefore the horizontal shift) should be $C=i+T/2$. The scaling factor should be the drift $A=d$, as this is the coordinates’ difference in the initial and end frames. Substitution of these three terms into Eq. (7) for $i=0$ (assuming tracking starts at the first frame) is required to find the last parameter $B$:${x}_{0}+\epsilon d=\frac{A}{1+{e}^{-B(0-C)}}+D$, substituting to

$${x}_{0}+\epsilon d=\frac{d}{1+{e}^{-B(-T/2)}}+{x}_{0}$$

$$\epsilon =\frac{1}{1+{e}^{BT/2}}$$

$${\epsilon +\epsilon e}^{BT/2}=1$$

$${e}^{BT/2}=\frac{1}{\epsilon }-1$$

$$B=2\mathrm{ln}(\frac{1}{\epsilon }-1)/T$$

Therefore, the full algorithm from $j=0$ to $j=T-1$ becomes:

8 $${x}_{\mathrm{new},j}={x}_{j}+\frac{d}{1+{e}^{-2ln(1/\epsilon -1)(j-T/2)/T}}$$

**Fig. 11:**
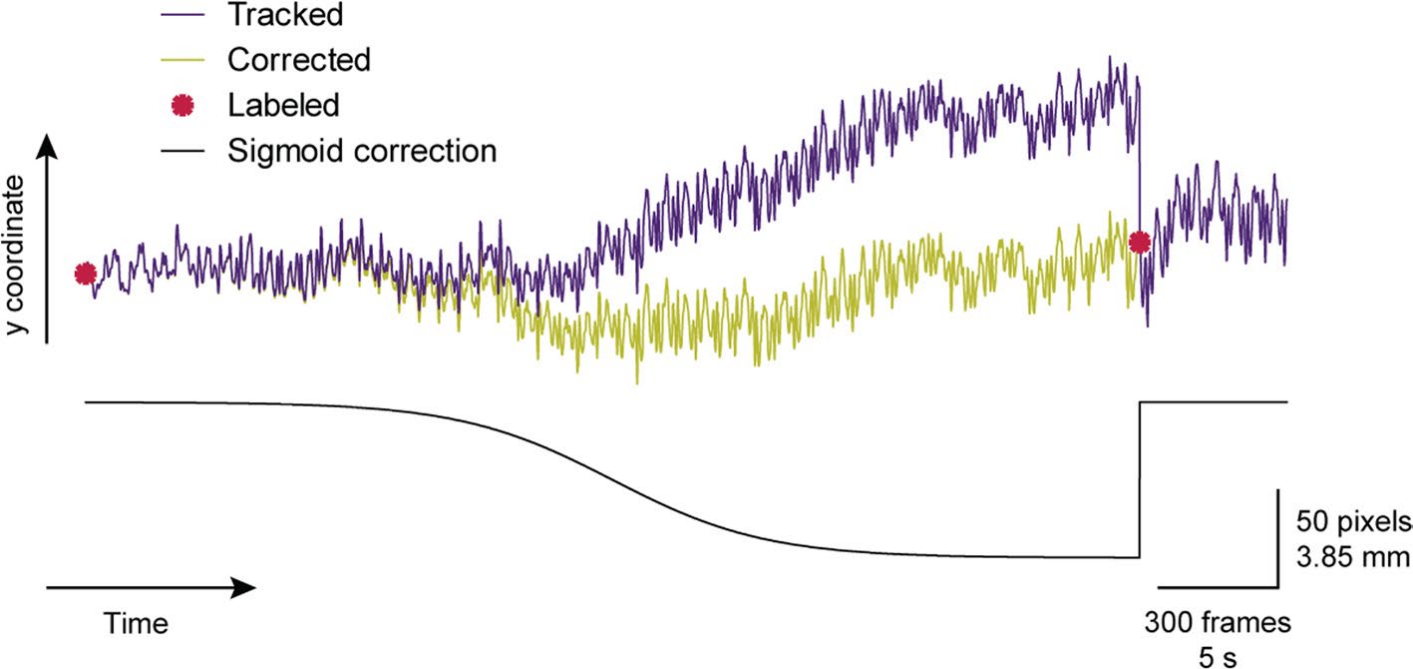
Sigmoid tracking correction visualized: this shows how STC can be applied to the example in Fig. 10. The purple line shows the tracked *y*-coordinate with LK starting at the first labeled point (left red point). The green line shows the path this point takes after applying sigmoid correction (black trace)

In the context of US tracking, this algorithm would be applied to tracking points for both $x$ and $y$ pixel coordinates. In Fig. 10, the tracking of the purple point drifts away from the desired feature to some higher *y*-coordinate. Figure 11 graphically shows an example of this algorithm in action. Once the labeled frame is reached, the tracker is reset to the desired coordinate, and the appropriate sigmoid function is added to compensate for the drift.

STC was used to improve the issue of drifting for all frame-to-frame trackers. Since STC requires labeled data to exist, it is important to understand how the quantity of labeled data improves tracking accuracy. 0–4 labeled frames per video were used for correction, called “correction frames,” which dictated at what frames the algorithm would reset the tracked points to their labeled location. As described earlier, each subject has 8 equally distributed labeled frames per video, so the chosen correction frames were also distributed evenly throughout. For 1 correction frame, labeled frame number 4 was used for correction. For 2 correction frames, labeled frame 3 and 6 were used for correction. For 3 correction frames, labeled frames 2, 4, and 6 were used for correction. And for 4 correction frames, labeled frames 1, 3, 5, 7 were used for correction.

#### Reversed tracking correction (RTC)

STC is a heuristics-based technique that requires labeled frames throughout the video of interest and is used after tracking is complete. In the scenario where no labeled point data is available, a method called reversed tracking correction (RTC) is proposed.

Given a set of initial points, one can assume in the worst scenario that drifting will increase error throughout the course of a video. Under this assumption, if the video is reversed with a set of initial points, running the same frame-to-frame tracker will also lead to drifting with increasing error. Using this information, a given tracker is applied normally to the original video starting at the start frame’s labeled points, and the tracked points are stored as the forward path *P*_f_. Then, the tracker is applied to the reversed video starting at the end frame’s labeled points, and tracked points are stored as the backward path* P*_b_.

${P}_{\mathrm{f}} ,{P}_{\mathrm{b}} (Nxpx2)$ → path forward, path backward (*x*, *y* coordinates).

${t}_{\mathrm{i}},{t}_{\mathrm{e}}$ → initial frame, end frame

$N,p$→number of frames, $p$ number of points.

RTC averages the two paths $({P}_{\mathrm{f}} +{P}_{\mathrm{b}})/2$, returning a new tracked path that includes an equal contribution from both.

#### Reversed sigmoid tracking correction (RSTC)

STC is useful when many frames throughout a video are labeled. RTC can be useful when only the start and end frames of a video are labeled. Reversed sigmoid tracking correction (RSTC) combines ideas from both methods to potentially improve accuracy given the same minimal information as in RTC. However, instead of an equal contribution from both paths, RSTC creates a new path that is more influenced by the forward path near the start of the video, and more influenced by the backward path near the end of the video. To do so, it scales the forward path by a sigmoid function with a value near 1 at the start frame, and a value near 0 at the end frame, and repeats this in reverse for the backward path. The sigmoid function parameters are found using a similar process as in Eq. (8):

9 $${{s}_{\mathrm{f}}=\frac{1}{1+{e}^{-B(x-C)}}}, s_{\mathrm{b}}=\frac{1}{1+{e}^{B(x-C)}}$$

where $$B=2\mathrm{ln}(\frac{1}{\epsilon }-1)/({t}_{e}-{t}_{i})$$ and $$C=({t}_{i}+{t}_{e})/2$$

$${P}_{\mathrm{fs}}={s}_{\mathrm{f}}{P}_{\mathrm{f}}, {P}_{\mathrm{bs}}={s}_{\mathrm{b}}{P}_{\mathrm{b}}$$(10)

$${P}_{T}={P}_{\mathrm{fs}}+{P}_{\mathrm{bs}}$$

**Fig. 12:**
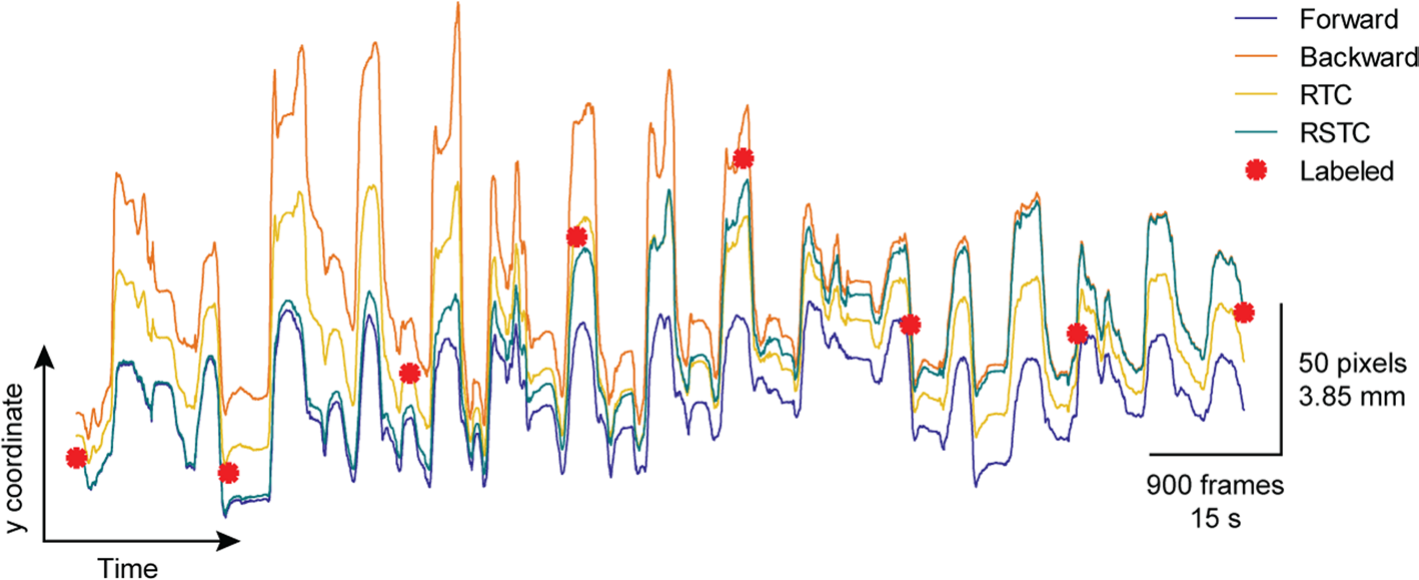
Reversed tracking correction and reversed sigmoid tracking correction visualized: visual of RTC and RSTC using the *y*-coordinate of an example point. The purple line represents the path of the point tracked forward in time. The orange line represents the path of the point tracked backward in time. The yellow line is the averaged path from RTC. The green line is the sigmoid-scaled path from RSTC. The red points are the human labeled points

To visualize the difference between RTC and RSTC, the path of an example point’s *y*-coordinate while tracking is shown in Fig. 12.

STC, RTC, and RSTC attempt to decrease the drifting errors of frame-to-frame trackers, but these heuristics-based approaches do not necessarily fix full tracking failures or tracking subtle movements. In an attempt to incorporate training using the labeled frames, an existing method of tracking using Convolutional Neural Networks (CNNs) will be described next in “DeepLabCut (DLC)”.

### DeepLabCut (DLC)

DLC is an open-source software used typically for markerless feature tracking of animals [31]. It uses the feature detector architecture from DeeperCut [45], a high-accuracy human pose-estimation algorithm that includes a network pretrained on more than 1.4 million images. This allows it to use a small number of training images (in order of a few hundred) to achieve human-level accuracy. DLC combines the pretrained ResNet encoder (typically used for object recognition with excellent performance) and deconvolutional layers (used to produce spatial probability densities) into a robust tracking CNN [31].

While DLC has been used extensively to track animal and human features during movements, its ability to track features in US videos has been minimally explored. [46] used DLC to track the upper surface of the tongue and compared it to other US contour estimators, concluding that DLC requires significantly less training data to perform with the same level of accuracy. [47] used DLC to track the gastrocnemius muscle–tendon junction, observing the morphology of the lower leg longitudinally. In addition, [48] used DLC as a general CNN to track hyoid kinematics during swallowing, comparing its accuracy to other manual and automatic hyoid-specific trackers. All related applications of this package not only focus on tracking different anatomies than those studied in this paper, but they also focus on tracking a singular feature. In this paper, DLC is used to simultaneously track features in soft tissue, bone, and adipose while comparing its accuracy with non-CNN trackers.

DLC has a Python toolbox [49] used to facilitate the manual steps involved with selecting videos, labeling points, and training and evaluating the network. The toolbox can be accessed via any text editor or its own GUI targeted towards those without programming experience. As described in “Human labeling”, both subject groups were labeled differently to tackle how tracking various features (Group 1) varied from tracking specific features across subjects (Group 2). Subjects in Group 1 had 8 equally spaced labeled frames per video, and half of those labeled frames across all videos were chosen as training data. Subjects in Group 2 had 10 equally spaced labeled frames per video, and 80% of the videos were chosen as training data. The rest of the frames and videos for these groups was chosen as testing data on which the DLC models would be evaluated. The manually labeled data of both Group 1 and Group 2 was restructured into the appropriate file types for DLC to use for training. The training data was augmented using the *imgaug* method (https://github.com/aleju/imgaug) and a 50-layer ResNet network was re-trained using this data for 500 k iterations, where error commonly plateaus [46] for ResNet50.

Although this series of steps typically performs visually well, DLC has an additional feature that checks the results for outlier frames by observing large changes in feature trajectories. Depending on the accuracy when manually labeling and the robustness of that particular network, the 144 originally labeled points might not have sufficiently eliminated these outliers, so the option to label additional frames was provided. However, in order to establish a standard benchmark in which all trackers used the same labeled frames, this outlier-detection feature was not used.

### Measuring accuracy and speed

In order to compare the accuracy of all algorithms, the error of interest was measured as the Euclidean distances between the ground truth labeled points and the center of the tracker’s estimated bounding boxes. This was done both before and after correction to compare both the accuracy between frame-to-frame algorithms (like OpenCV trackers) and trained deep networks (like DLC) given a specified number of labeled frames. These same distance metrics were used to compare tracking accuracy depending on the type of movement performed, or the number of correction frames used.

Next, to understand the impact of cumulative drifting, 8 copies of each labeled video were made and cropped to have an increasing number of frames (*N*)—in this case, *N* = 200, 400, … 1400, 1600. Each copy was then reversed and concatenated to the end of the non-reversed copy to create videos with 2*N*-1 frames as if each copy was mirrored onto itself. Then, from the starting frame and its labeled points, each tracker was applied to these copies, and the distances between the points at the start and end frames were measured. Since these copies are mirrors of themselves, the start and end frames were the same, so an ideal non-drifting tracker would return an error of 0. The larger the error between the points, the worse the drifting for that tracker.

Finally, to analyze the speed of these various methods, the time to completion of each algorithm was determined. A computer with a 3 GHz CPU and 64 GBs of RAM was used (Intel i9-9980 XE) to run all frame-to-frame trackers, and only one tracking process ran on it at a time. Another computer’s 1860 MHz GPU (NVIDIA GeForce RTX-3090 Ti) was used to run all DLC processes, as the DLC GitHub (https://deeplabcut.github.io/DeepLabCut/) explains code will run an order of magnitude faster using a GPU.

## Supplementary Information

**Additional file 1: Supplementary data and results. **.

**Additional file 2. Data presented in the figures, along with individual data points are provided in this excel file.**

## Data Availability

Data presented in the figures are provided as supplementary material.

## Abbreviations

US: Ultrasound
LK: Lucas–Kanade
MOSSE: Minimum output sum of squared error
KCF: Kernelized correlations filter
CSRT: Channel and spatial reliability tracker
DLC: DeepLabCut
STC: Sigmoid tracking correction
RTC: Reversed sigmoid correction
RSTC: Reversed sigmoid tracking correction
MVC: Maximum voluntary contraction
CNN: Convolutional neural network
