# Supplementary material for: A comparison of point-tracking algorithms in ultrasound videos from the upper limb
Source: Biomed Eng Online. 2023 May 24;22:52. doi: 10.1186/s12938-023-01105-y (PMC10207829; doi:10.1186/s12938-023-01105-y)
Supplement: Supplementary file 1 — Additional file 1: Supplementary data and results. . [file 12938_2023_1105_MOESM1_ESM.docx]

**SUPPLEMENT**

**A comparison of point-tracking algorithms in ultrasound videos from the upper limb**

Uriel Magana-Salgado ^1,2,*^, Praneeth Namburi ^3,4,5,*^, Micha Feigin-Almon ^1^, Roger Pallares-Lopez ^1,2^, Brian Anthony ^1,3,4^

^1^ Department of Mechanical Engineering, MIT, Cambridge, MA 02139, USA.

^2^ Mechanical Engineering Graduate Program, MIT, Cambridge, MA 02139, USA.

^3^ Institute for Medical Engineering and Science, MIT, Cambridge, MA 02139, USA.

^4^ MIT.nano Immersion Lab, MIT, Cambridge, MA 02139, USA.

*Co-first authors

^5^ To Whom Correspondence Should Be Addressed:

Dr. Praneeth Namburi

Institute for Medical Engineering and Science

77 Massachusetts Ave, 12-3211

Massachusetts Institute of Technology, Cambridge, MA 02139, USA.

praneeth@mit.edu

1. Supplement

# Bounding Box Sizes Affect Tracking Accuracy

OpenCV trackers are initialized by drawing a box bounding the region of interest. These box sizes can also alter tracker accuracy and speed, so we compared errors across all trackers with RSTC using three different box sizes- 100x100 pixels, 50x50 pixels, and 15x15 pixels - as shown in Supplementary Figure 1a.


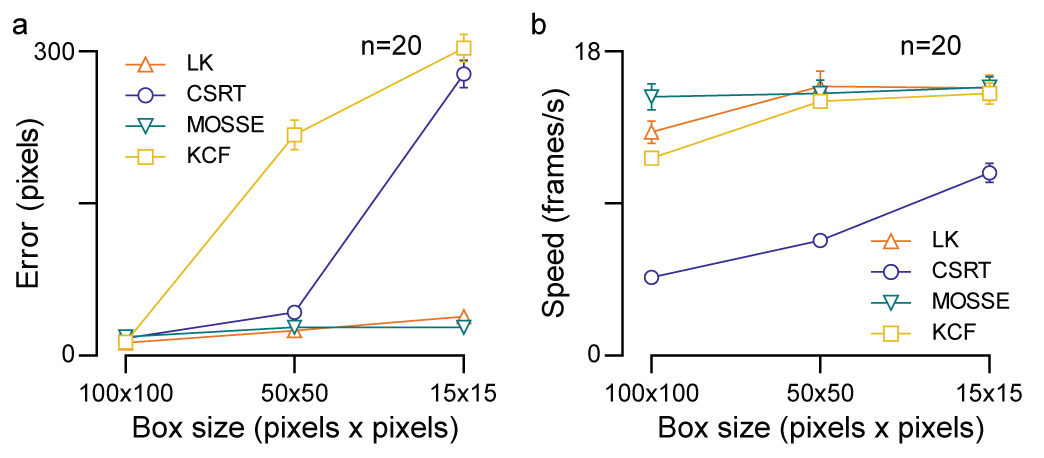


## **Supplementary Figure 1 – Bounding Box Size Effects on Accuracy**: (a) Accuracy of frame-to-frame trackers using various bounding box sizes. (b) Speed of frame-to-frame trackers using various bounding box sizes. Error bars refer to SEM across the 20 subjects for this experiment.

KCF performed well when tracking with a large box (100 pixels), but it consistently ran into tracking failures with a mid-sized or small box (50 pixels, 15 pixels). CSRT similarly performed poorly with a small box, but achieved a much smaller error than KCF using mid-sized boxes (50 pixels). LK’s error also increased with box size, but to a much lesser extent than KCF and CSRT. MOSSE resisted failures and maintained a relatively low error for all box sizes. Generally, in order to obtain the lowest error values when tracking, using a large box size was optimal.

There are a few distinctions to make when comparing the quality of the trackers aside from the quantitative error metric found above. As shown in Supplementary Figure 1a, KCF with a 100x100 pixel box had one of the lowest accumulated errors compared to its other frame-to-frame tracker counterparts, but the total average error for this tracker when using a 15x15 pixel box increased by an order of magnitude. When inspecting the tracking quality, we noticed this high error was due to tracking failures in which we reset the boxes to the origin coordinate. A large box size is recommended to prevent these failures from occurring.

Next, we calculated the speed of each tracker (in frames per second) using these bounding box sizes. CSRT was consistently the slowest of the algorithms while the MOSSE tracker did not significantly change speed as its box size changed. The speed of LK and KCF both plateaued as box size decreased. Supplementary Figure 1b shows that one of the biggest caveats of using a larger box for improved tracking is computational cost. However, due to the substantial error increases relative to the minimal increases in speed for all trackers, it is still recommended that a larger box size is used. Although slower, it can prevent them from potential tracking failures and improve accuracy significantly.

# DLC model certainty does not affect model application time to videos

Computational speed was also important for DLC. The time to apply the models to each group of videos was found, and the speed was calculated by average number of frames processed per second. Supplementary Figure 2a shows that once a model is trained, applying that model to track points in any video has similar computational cost regardless of the model accuracy in tracking or the number of iterations that model took to train.


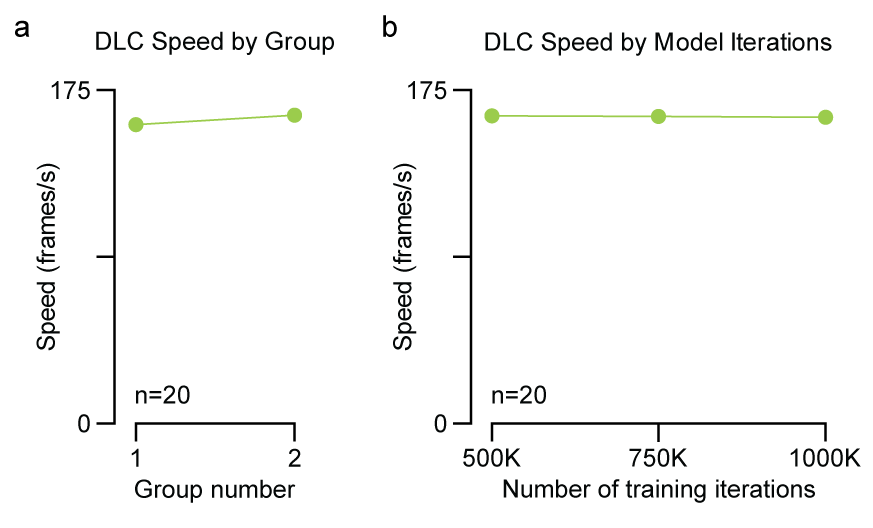


## **Supplementary** **Figure 2 – DLC speed during tracking**: (a) Speed of applying the two respective DLC models to Groups 1 and 2. (b) Speed of applying the 3 DLC models trained with increasing training iterations to Group 2.

More details about the computational power used to run these various algorithms are detailed in section “Measuring accuracy and speed” of the main text, as DLC runs at a much higher speed on a computer with a GPU.

# Variance in Human Labeling

Up to now, our error values have used one set of labels as ground truth; however, as discussed in section “Approaching drifting correction using three novel methods” of the main text, labeling often involved rough estimates of feature location. To understand human variability when labeling, we relabeled the points two more times and found their average distance to the original labels to be 12.6±0.9 pixels. This error was high but expected due to the prominent deformation of skeletal tissue and fascia. Supplementary Figure 3 was made from the data in Figure 5 of the main text, displaying how the various algorithms can partly be guided by the points selected as ground truth.


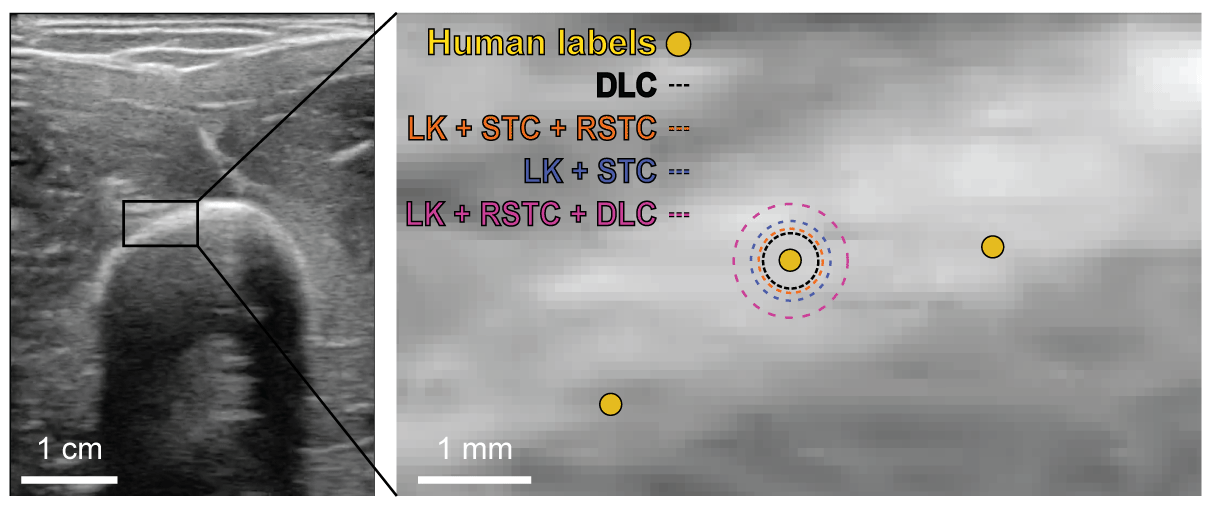


## **Supplementary** **Figure 3 – Human variance in labeling process**: Highlights how variability of human labeling around ambiguous features in US can affect tracking and respective error values.

This shows a key benefit of using these trackers in their ability to accurately follow labeled points, but also highlights the importance of having clear, distinct, ubiquitous features that are carefully labeled.

# DLC post-processing analysis

Post-processing algorithms can reduce the jitter contained in the DLC tracking signal, and at the same time, potentially improve the position of tracked points in low-likelihood frames by introducing temporal information or known dynamics of pixels. To evaluate this fact, we integrated a Kalman filter and a low-pass filter to DLC and compared the results.

A Butterworth low-pass filter with a cut-off frequency of 10 Hz was implemented using the Scipy Python library. Similarly, a Kalman filter that assumed constant pixel speed dynamics between frames was derived and implemented.

## **
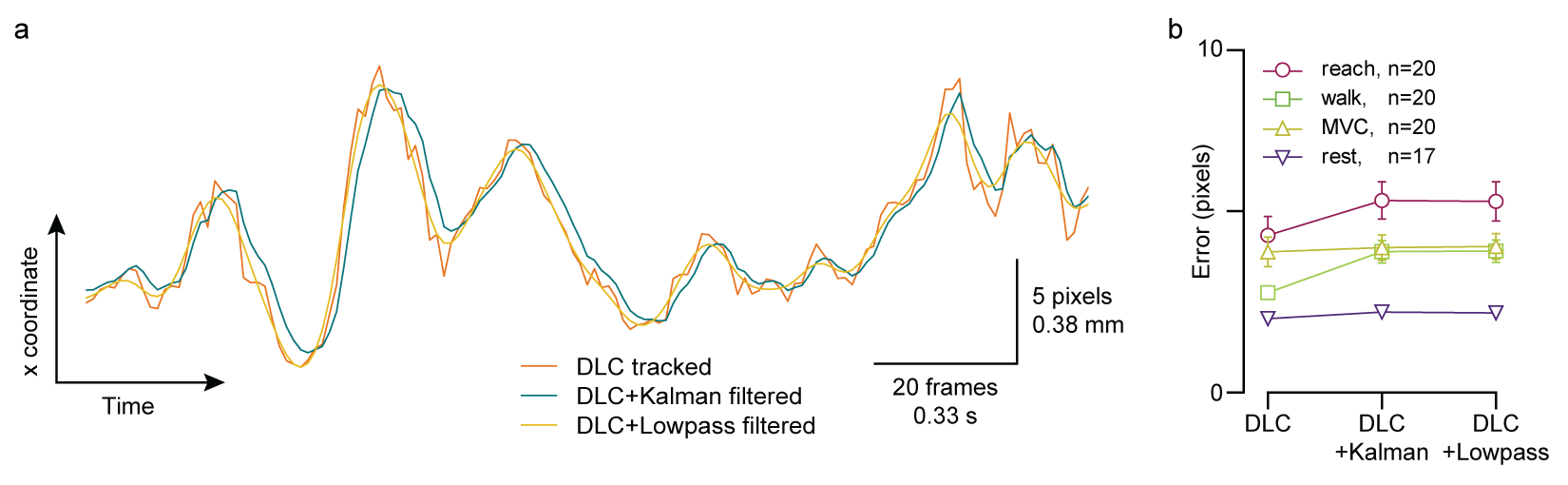
Supplementary** **Figure 4 – DLC post-processing results**: (a) Illustrative example of the effects of the low-pass filter and the Kalman filter to the raw DLC results. (b) Accuracy of DLC and DLC plus post-processing algorithms for each studied task.

From visual inspection, one can appreciate that both Kalman and low-pass filter reduce the high-frequency content of the DLC tracking, and smooth out local maxima and minima (Supplementary Figure 4a). While the low-pass filter achieves so by removing all components above the cut-off frequency, Kalman compares the pixel dynamic model with the given data (DLC tracking), and obtains a posterior pixel position with reduced high frequency content assumed to be noise. However, when sudden changes of pixel position appear, Kalman generates a result with lag, which is a consequence of the simplified dynamics of pixel motion.

When comparing the errors from labeled frames (Supplementary Figure 4a), no significant changes are appreciated once the low-pass or Kalman filter are used (one-way ANOVA: reach - *F* = 1.1, *p* = 0.34, MVC - *F* = 0.042, *p* = 0.96, rest - *F* = 0.41, *p* = 0.67). Even for the walking motion, the errors appear to be significantly worse (one-way ANOVA: walk - *F* = 5.6, *p* = 0.0059).

Therefore, the best post-processing approach to improve DLC, and in general any tracking algorithm, remains an open question. Several options are available, and a range of subtle changes can be implemented to address a specific tracking task. For instance, we believe that an improved dynamic model of pixels, including information of the acceleration, would improve the Kalman filter performance. For all these reasons, further research is essential for understanding the characteristics of each specific tracking task, and consequently implementing the most appropriate post-processing algorithm, which is beyond the scope of this work.
